# Response to the letter to the editor “The posterior question mark incision for decompressive hemicraniectomy”

**DOI:** 10.1007/s00701-022-05228-4

**Published:** 2022-05-02

**Authors:** Michael Veldeman

**Affiliations:** Department of Neurosurgery, RWTH Aachen University Hospital, Pauwelsstrasse 30, 52074 Aachen, Germany

We thank the authors for their interest in our work and the time and effort they took to comment on our results [3]. The skin incision used for unilateral decompressive craniectomy constitutes the larges scalp flap performed in current neurosurgical practice. This skin flap is simply, by merit of size, prone to problems of wound infection and wound healing. It is not helpful that these patients are mostly bedridden, causing compression of a partially devascularized skin flap. The problem becomes even more relevant during cranioplasty when the avascular bone flap is reinserted. Cranioplasty failure due to infection is observed in around 1/3 of procedures [1]. The incentive for an incision ending behind the ear is the preservation of the superficial and deep temporal arteries and their branches. In our series of 186 cranioplasty surgeries, the posterior question mark incision was associated with a reduced rate of cranioplasty failure due to infection [2]. We attribute this effect to the better vascularization of the skin flap and a potential better immune response to pathogen contamination.

In essence, nor shape neither size of the planned removed bone flap should differ between a standard question mark incision and a retroauricular ending one. The incision is carried downward up to the level of the mastoid notch, but the bony removal by the craniotomy obviously remains always superior of the transverse sinus and floor of the petrous bone. Extensive pneumatization of the temporal bone up into the temporal squama is rare, but potentially in those anatomical variations, bony air cells could be opened accidentally. In this type of craniotomy, we have not encountered accidental opening of temporal boney air cells in connection with the mastoid. As in the articles that were referred to in your comment, pneumatization of the temporal bone posteriorly to the mastoid up to the lambdoid suture increases the risk of cerebrospinal fluid fistulae, when performing a craniotomy below the superior nucheal line (and transverse sinus) for example in a typical retrosigmoid approach.

We have documented one case during which the external auditory canal was indeed opened when the skin flap was retracted too forcefully downward. At least, this patient presented with otohemorrhea, and after careful inspection, there was injury to the epithelial lining of the cartilaginous meatus. An outer marking of the external meatus could help localizing its position during dissection but might prove difficult to achieve accurately as the folding of the flap over the ear hinders appreciation of such a marking. Once the temporal muscle is deflected caudally, one usually gets a good impression where the posterior ending of the zygomatic process is. Staying above the zygomatic process will prevent to caudal dissection, as the external auditory canal is always below it.

The problem of compression of the retroauricular wound in bed ridden patients is relevant. Care must be taken to frequently reposition patients and intensive care nursing staff has to be made aware of this issue. As pressure ulcer prevention in bed-ridden patients dictates repositioning anyway, this does not entail a relevant increase of workload for nursing staff. The problems we encountered with the classical trauma flap were almost exclusively located at pressure point, mainly in the occipital region. Despite the possibility of more compression of the posterior extension of the wound behind the ear, we have achieved better result with the retroauricular incision. Again, we believe that this is due to superior vascularization compared to a trauma flap.

It should be emphasized that in our opinion, this style of incision does not require extra skill. Also, as the incision lays completely within hairlines, the esthetical outcome after cranioplasty is similar to a preauricular incision. As the authors pointed out, the posterior question-mark incision is a valuable alternative to the standard trauma flap, and therefore, we strongly encourage external validation of our positive results.
